# The Endothelium as a Therapeutic Target in Diabetes: A Narrative Review and Perspective

**DOI:** 10.3389/fphys.2021.638491

**Published:** 2021-02-23

**Authors:** Jose A. Adams, Arkady Uryash, Jose R. Lopez, Marvin A. Sackner

**Affiliations:** ^1^Division of Neonatology, Mount Sinai Medical Center, Miami Beach, FL, United States; ^2^Department of Research, Mount Sinai Medical Center, Miami Beach, FL, United States; ^3^Department of Medicine, Mount Sinai Medical Center, Miami Beach, FL, United States

**Keywords:** pulsatile shear stress, whole body periodic acceleration, enhanced external counter pulsation, whole body vibration, passive jogging device, exercise, diabetes, nitric oxide

## Abstract

Diabetes has reached worldwide epidemic proportions, and threatens to be a significant economic burden to both patients and healthcare systems, and an important driver of cardiovascular mortality and morbidity. Improvement in lifestyle interventions (which includes increase in physical activity via exercise) can reduce diabetes and cardiovascular disease mortality and morbidity. Encouraging a population to increase physical activity and exercise is not a simple feat particularly in individuals with co-morbidities (obesity, heart disease, stroke, peripheral vascular disease, and those with cognitive and physical limitations). Translation of the physiological benefits of exercise within that vulnerable population would be an important step for improving physical activity goals and a stopgap measure to exercise. In large part many of the beneficial effects of exercise are due to the introduction of pulsatile shear stress (PSS) to the vascular endothelium. PSS is a well-known stimulus for endothelial homeostasis, and induction of a myriad of pathways which include vasoreactivity, paracrine/endocrine function, fibrinolysis, inflammation, barrier function, and vessel growth and formation. The endothelial cell mediates the balance between vasoconstriction and relaxation via the major vasodilator endothelial derived nitric oxide (eNO). eNO is critical for vasorelaxation, increasing blood flow, and an important signaling molecule that downregulates the inflammatory cascade. A salient feature of diabetes, is endothelial dysfunction which is characterized by a reduction of the bioavailability of vasodilators, particularly nitric oxide (NO). Cellular derangements in diabetes are also related to dysregulation in Ca^2+^ handling with increased intracellular Ca^2+^overload, and oxidative stress. PSS increases eNO bioavailability, reduces inflammatory phenotype, decreases intracellular Ca^2+^ overload, and increases antioxidant capacity. This narrative review and perspective will outline four methods to non-invasively increase PSS; Exercise (the prototype for increasing PSS), Enhanced External Counterpulsation (EECP), Whole Body Vibration (WBV), Passive Simulated Jogging and its predicate device Whole Body Periodic Acceleration, and will discuss current knowledge on their use in diabetes.

## Introduction

Diabetes is a worldwide epidemic, with a global prevalence of over 425 million. Diabetes accounts for nearly 9.4% of the United States population with an estimated cost of over $300 billion annually (Willey et al., 2018). Globally diabetes will have an economic burden of over $2.1 trillion by 2030 (Bommer et al., 2018). Diabetes is a public health threat and one of the most important risk factors for cardiovascular disease. Type 2 diabetes (T2D), accounts for 90% of all persons with diabetes (American Diabetes, 2020). An absolute or progressive loss of adequate insulin secretion and insulin resistance, leads to the cardinal manifestation of diabetes which is hyperglycemia. Hyperglycemia, leads to a host of cellular derangements which induce free radial generation leading to oxidative stress (Yaribeygi et al., 2019), endothelial dysfunction (Meza et al., 2019; Tran et al., 2020), and cardiomyocyte dysfunction leading to heart failure (Singh et al., 2018; Nakamura and Sadoshima, 2020). Further, hyperglycemia impacts the immune system with suppressed cytokine production, defects in leukocyte recruitment, and dysfunction in neutrophil, macrophages, and natural killer cell (Berbudi et al., 2020). Key determinants of the effects of diabetes on both cardiomyocytes and vascular endothelial cells involve decrease in nitric oxide (NO) bioavailability, increase reactive oxygen species (ROS), and inflammation (Goligorsky, 2017; Filardi et al., 2019; Joubert et al., 2019; Trigglea et al., 2020) as well as deranged calcium homeostasis (Murtaza et al., 2019). It would be overly simplistic to only recognize hyperglycemia as the culprit in diabetes, but insulin resistance also induces signaling pathways which are involved in the micro and macro vascular pathologies which are seen in diabetes (Petersen and Shulman, 2018).

A salient feature of diabetes, is endothelial dysfunction. Endothelial dysfunction is characterized by a reduction of the bioavailability of vasodilators, particularly nitric oxide (NO), and/or an increase in endothelium-derived contracting factors (Hadi et al., 2005; Sena et al., 2013; Cyr et al., 2020). The resulting imbalance leads to an impairment of endothelium-dependent vasodilation, which is the functional characteristic of endothelial dysfunction. Endothelial dysfunction is the hallmark of various cardiovascular diseases including, heart failure, the precursor to atherosclerosis, early stages of Alzheimer disease, and micro and macrovascular disease (Jia et al., 2018; Takeda et al., 2020). Additionally, cellular derangements in diabetes are related to dysregulation in Ca^2+^ handling particularly in cardiomyocytes. The latter mainly occurs due to defects in sarcolemmal Na^+^/K^+^ ATPase, Na^+^/Ca2^+^ exchange, Na^+^/H^+^ exchange, Ca^2+^-channels and Ca^2+^-pump activities as well as changes in sarcoplasmic reticular Ca^2+^-uptake and Ca^2+^-release processes. These alterations lead to intracellular Ca^2+^ overload (Zarain-Herzberg et al., 2014; Singh et al., 2018).

To understand the concept of utilizing pulsatile shear stress (PSS) as a therapeutic intervention one must understand the endothelium. The endothelium is a monolayer of endothelial cells (EC) which covers the luminal surface of arteries, veins, capillaries, and the heart. The EC surface area covers 3000–6000 m^2^ of the human body. The EC is heterogeneous, numerous in location and vast in mediators (Davies, 1995; Aird, 2003; Ohura et al., 2003; Bryan et al., 2014; Firasat et al., 2014; He et al., 2020), and adaptive as to its response to stimulus (Resnick et al., 2003; Chien, 2007; Himburg et al., 2007; Zhang and Friedman, 2012, 2013). The EC is vital as both a transducer of a multitude of mechanical and biochemical signals, as well as an effector cell. The mechanical sensing capabilities of EC are governed by physical forces (tangential, radial, axial) and local blood flow patterns (Gordon et al., 2020; Roux et al., 2020). Blood flow produces mechanical frictional forces which produce endothelial shear stress, in addition with each contraction of the heart a pulse is added to the circulation due to the mechanical contraction of the heart producing PSS, pulsatile shear stress promotes EC homeostasis and vascular health (Kruger-Genge et al., 2019; Gordon et al., 2020). In addition to frequency of pulsatility, flow patterns within the vasculature are also important. Whereas laminar shear stress (tangential to the surface of the blood vessel) produces a cytoprotective EC phenotype (non-atherogenic, non-inflammatory), oscillatory shear (OS, as seen in areas of vascular stenosis or bifurcation) and low shear stress have been shown to be inflammatory and atherogenic (Qiu and Tarbell, 2000; Kadohama et al., 2007; Laughlin et al., 2008; Zhou et al., 2014; Ajami et al., 2017; Wang et al., 2019; Souilhol et al., 2020). Endothelial cells are interconnected by cellular junctions that confer selective permeability. The EC’s strategic location to sense and respond to both hemodynamic changes as well as circulating biochemical signals, makes it a formidable regulator of the human body’s general homeostasis. The input of the EC thus relates to both mechanical (shear stress) as a mechanosensor and biochemical signals as a chemosensor (Campinho et al., 2020; He et al., 2020). The output of the EC is involved in a myriad of pathways, which includes vasoreactivity, paracrine/endocrine function coagulation and blood fluidity, inflammation, barrier function, vessel growth, and formation (vasculogenesis, angiogenesis, arteriogenesis) (Trigglea et al., 2020).

The endothelium mediates the balance between vasoconstriction/relaxation by secreting mediators, such as endothelin-1 and thromboxane-A2 (vasoconstriction) and nitric oxide (NO), prostacyclin, and endothelium-derived hyperpolarizing factor (EDHF) (vasodilatation). This summary will focus our attention on the most important vasoactive/signaling molecule endothelial derived nitric oxide (eNO), and antioxidants. Nitric Oxide (NO) is a gas which is important in signal transduction, protein S-Nitrosylation, and critical for vasodilation (Forstermann and Sessa, 2012; Duran et al., 2013; Ghimire et al., 2017; Farah et al., 2018). NO is produced by oxidation of L-arginine to L-citrulline utilizing the enzyme nitric oxide synthase (NOS) and tetrahydrobiopterin (BH4) as cofactor to produce NO. There are three nitric oxide synthases; (a) Endothelial derived nitric oxide synthase (eNOS) produced in most tissues but primarily in the endothelium. eNOS is constitutively expressed and calcium dependent. eNOS produces nanomolar amounts of NO. (b) Inducible nitric oxide synthase (iNOS), which is not constitutively expressed. iNOS produces large quantities of NO, usually produced by macrophages, and inflammatory cells, and (c) Neuronal nitric oxide synthase (nNOS) which is found mostly in both neuronal and cardiovascular tissue, has a role in neuronal signal transduction and chronotropicity of the heart (Ziolo et al., 2008; Forstermann and Sessa, 2012). eNO is an important molecule, which is produced in response to pulsatile shear stress. In classic and elegant experiments performed by Hutchenson et al., they found that NO [previously called endothelium derived relaxing factor (EDRF)] is produced by endothelial cells as a function of pulsatility with optimum frequency of pulsation of 2–8 Hz (120–480 cpm) (Hutcheson and Griffith, 1991). The direct response of eNOS activation and NO production to PSS in these experiments and others, have clearly shown that PSS is important for endothelial homeostasis. eNO is critical for vasorelaxation, increasing blood flow, and an important signaling molecule that downregulates the inflammatory cascade (Albrecht et al., 2003; Cirino et al., 2003; Kolluru et al., 2010; Forstermann and Sessa, 2012). The concept of modulating the endothelium using PSS via non-invasive methods, is thus an attractive method to modify or prevent various chronic diseases including diabetes. The prototype of such a model is exercise. Unfortunately, comorbidities (obesity, heart failure, peripheral vascular disease, stroke, and physical or cognitive limitations) which coexist in the diabetic population and those at risk, make exercise difficult or impossible. Simple methods to induce PSS are highly desirable to maintain endothelial homeostasis, promote eNO for cell signaling and combat downstream effects of diabetes at the cellular level. This review will first focus on exercise, the prototype for non-invasive methods for PSS, and discuss Enhanced External Counterpulsation (EECP), Whole Body Vibration (WBV), and Passive Simulated Jogging Device (JD) and its predicate device Whole Body Periodic Acceleration (WBPA) within the context of their beneficial effects on diabetes.

## Interventions to Achieve Pulsatile Shear Stress and Search Methods

Pulsatile Shear Stress, occurs naturally in the human body. Each contraction of the heart adds pulsations to the circulation, producing a frequency of pulsatility of 1 to 2 Hz and additional pulsations to the circulation beyond this frequency increases eNO bioavailability (Hutcheson and Griffith, 1991; Stefano et al., 2001).

**FIGURE 1:**
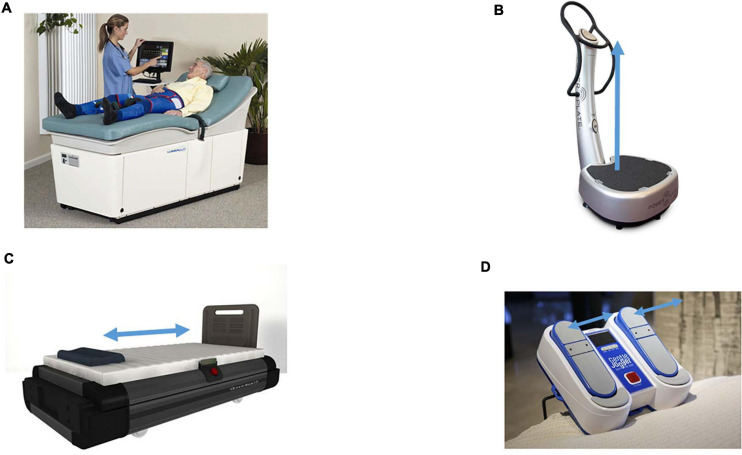
Devices which produce sustained pulsatile shear stress. Devices which produce sustained pulsatile shear stress (PSS). **(A)** Enhanced External Counter Pulsation (EECP). External synchronized compression of the lower extremities via bladder like attachments on the lower extremities, the compressions are timed to diastole. Picture is The Lumenair^TM^ System EECP^®^ both are registered trademarks of VasoMedical, Inc. (Courtesy of VasoMedical, Plainview, NY, United States. **(B)** Whole Body Vibration (WBV), provides vertical vibration, with the vector of movement in the foot to head direction (+ Gz). The pictured device is Power Plate^®^ my5^TM^ (Performance Health Systems, LLC, Northbrook, IL, United States). **(C)** Whole Body Periodic Acceleration (WBPA) moves the supine body in a head to foot sinusoidal motion (± Gz). The pictured device is WBPA from Non-invasive Monitoring System (NIMS, Miami, FL, United States). **(D)** Passive Simulated Jogging Device (JD) is a passive motorized device which produces alternating movement of the foot simulating jogging introducing pulsations to the body. The device can be used in the seated or supine posture. The pictured device is the Gentle Jogger^®^ (Sackner Wellness Products, Miami, FL, United States). Blue arrows represent the acceleration motion of each device.

This review will focus on four non-pharmacologic methods for sustained shear stress, which increase pulsatile shear stress to the vascular endothelium and have been shown to confer a protective role in diabetes and other diseases; Exercise, EECP, WBV, and Whole Body Periodic Acceleration (WBPA/pGz), or Passive Jogging Device (JD) Figure 1.

In addition to the above interventions a literature search was performed using PubMed and EMBASE Databases. The first search involved key terms; Pulsatile Shear Stress or Shear Stress (457 articles), two additional methods were identified. Individual Shear Rate Therapy (ISRT, a method which is similar to EECP) and Pulsed High Intensity Ultrasound (a method not used as therapy in diabetes). The following key terms were then used; (a) diabetes or diabetes mellitus, or diabetic, and exercise or exercises or exercise therapy. (b) enhanced external counterpulsation or counterpulsation, or external counterpulsation, and diabetes or diabetes mellitus, or diabetic (c) whole body vibration or body vibration, or human body vibration and diabetes or diabetes mellitus, (d) passive simulated jogging or passive jogging or simulated jogging and diabetes or diabetes mellitus, or diabetic (e) whole body periodic acceleration or periodic acceleration and diabetes or diabetes mellitus, or diabetic. The search was limited to randomized controlled trials, review articles, or meta-analyses, with studies limited to those in English with human participants. Titles and abstracts were reviewed for relevant information with regards to the aforementioned sustained pulsatile shear stress interventions in relation to diabetes. Search dates January 1990 to November 2020.

## Exercise

### Exercise for Pulsatile Shear Stress

Exercise is the prototype of a non-invasive method to increase pulsatile shear stress. Shear stress (SS) and/or circumferential wall stress (stretch) are the primary signals produced by exercise. SS levels in the arteries of humans during exercise are in the range that produces beneficial changes in endothelial phenotype (Laughlin et al., 2008; Zhang and Friedman, 2013; Bender and Laughlin, 2015). The mechanisms responsible for the effects of exercise on the endothelial cell and vasculature are both primary and secondary in nature, and are in addition to benefits produced by changes in cardiovascular risk factors such as lipid profiles and blood pressure which occur in concert with the direct effects of arterial shear stress and mechanotransduction (Green and Smith, 2018). Exercise exerts direct effects on the vasculature via the impact of repetitive exposure to hemodynamic stimuli, such as shear stress and transmural pressure (Green et al., 2017; Wang et al., 2019).

Pulsatility, as well as shear stress, induce anti-inflammatory, and an atheroprotective phenotype in endothelial cells. Lifestyle modifications include both diet and exercise and have remained paramount in the management of both type 1 and type 2 diabetes. The primary goal of exercise in diabetes is to improve overall cardiovascular health, improve insulin sensitivity, and modify endothelial dysfunction produced by diabetes. Some of the additional features of exercise which benefit the management of diabetes include the reduction of oxidative stress. Pulsatile shear stress (PSS) is produced by exercise, and in particular running, jogging, or walking. As the individual walks or runs, additional pulses are added to the circulation from each step to the already existing cardiac pulsations. Thus, the cadence of walking or running provide additional pulsatility. Step frequency for both males and females recreational runners has been estimated between 163 and 169 steps per min (Clermont et al., 2019). This frequency is in addition to a baseline cardiac pulsatility of 60–100 beats/min, and therefore generates total pulsation close to 220–240 beats per min (3–4 Hz). Since the running cadence is not timed to the cardiac cycle one can expect frequencies in the range of 80–250 (1.3 to 4 Hz). It is not the intent of this narrative review to focus or discuss the various exercise strategies including resistance training or interval training which have been shown to produce beneficial effects on cardiovascular health, various excellent reviews have been published on this aspect (Kojda and Hambrecht, 2005; Ploughman, 2008; Wilson et al., 2016; Adams et al., 2017; Trigiani and Hamel, 2017; Fletcher et al., 2018; Yanai et al., 2018; Pinckard et al., 2019; Trinity and Richardson, 2019; Brown et al., 2020).

### Exercise and Diabetes

Under normal conditions glucose transporter-4 (GLUT-4) an intracellular protein is translocated to the cell membrane in response to insulin where it facilitates glucose uptake and utilization. In diabetes expression and function of GLUT-4 are compromised, leading to a reduction in glucose transport. Exercise increases GLUT-4 expression and glucose transport (Richter and Hargreaves, 2013). Additionally, exercise increases GLUT-4 translocation in healthy and diabetic models (Dohm, 2002; Henriksen, 2002; Ito et al., 2006). Exercise also induces pancreatic ß cells to increase insulin secretion activating insulin signaling pathway, upregulating GLUT-4 expression and enhancing intracellular energy metabolism (Stanford and Goodyear, 2014). Dysregulation of intracellular (Ca^2+^) homeostasis is another marker of diabetes, particularly in cardiomyocytes. Exercise improves the expression and activity of the Ca^2+^-ATPase (SERCA2a), and regulates Ca^2+^ levels via Ca^2+^ calmodulin- dependent protein kinase phosphorylation (Sturek, 2011; Guerrero-Hernandez and Verkhratsky, 2014). Hyperglycemia is also associated with chronic inflammation and oxidative stress. Hyperglycemia enhances the production of oxygen free radicals and apoptosis. Exercise reduces the production of ROS, and reduces oxidative stress damage, additionally exercise upregulates the expression and function of anti-oxidants enzymes superoxide dismutase, glutathione peroxidase and catalase (Seo et al., 2019), and “exerkines” (Yu et al., 2017). As a result of the above, exercise in diabetic patients; reduces overall cardiovascular disease burden, improves endothelial function, improves indices of cardiac dysfunction, reduces overall hyperglycemic time and reduces the long term index of poor glucose control (glycated hemoglobin, HbA1C) (Seo et al., 2019). Exercise is simple intervention, which reduces cardiovascular morbidity and mortality and positively correlates with beneficial health outcomes as they pertain to diabetes and other chronic illnesses. Exercise requires subject cooperation and comorbidities previously mentioned in the population with or at risk for diabetes may preclude patients from engaging and remaining on an exercise program. The following methods provide a stop-gap measure and potential alternatives for harnessing the beneficial effects of PSS.

## Enhanced External Counterpulsation

### Enhance External Counterpulsation (EECP) for Pulsatile Shear Stress

Enhanced external counterpulsation (EECP) is a non-invasive therapy which has its origin in the 1960s in a study by Kantrowitz (1953) related to an invasive mechanical delay of the aortic pressure pulse in an animal model, and by 1976 the device matured to its present form (Zheng et al., 1984). EECP involves pneumatic inflation of three pairs of cuffs, compressing the calves, thighs and buttocks in a sequential distal to proximal manner timed to early diastole followed by deflation before systole (Raza et al., 2017). The frequency of additional pulsations induced by EECP is a function of the subject’s intrinsic heart rate, since for every single diastole there is a corresponding inflation. EECP effectively doubles the heart rate pulsatility (120–200 beats/min or 2–3.3 Hz). In addition to pulsatile shear stress, EECP imparts circumferential stretch, and analysis of flow patterns under EECP, have also shown a less oscillatory flow (which is associated with endothelial dysfunction and pro-atherogenic phenotype) (Li et al., 2019). Original indications for EECP where related to angina due to coronary artery disease (CAD). The beneficial effects of EECP have expanded to peripheral artery disease, diabetes, erectile dysfunction, and possibly Alzheimer’s disease. Similar to exercise, EECP adds additional pulsations to the endothelium, which have been shown to induce production of nitric oxide (Akhtar et al., 2006), improve flow mediated vasodilation (FMD) endothelial function (Shechter et al., 2003; Braith et al., 2010), attenuate pro-inflammatory signaling pathways (Zhang et al., 2010; Martin and Braith, 2012) and improve quality of life (Jan et al., 2020) amongst others. The mechanisms for the effects produced by EECP appear to have both a central hemodynamic component with decreased afterload, increased coronary blood flow, and a peripheral effect (passive exercise) (Braith et al., 2012; Raza et al., 2017). In addition to the aforementioned, EECP also improves the oscillatory flow pattern in stenosed coronary arteries (Xu et al., 2020). The EECP device has a large footprint (1,861,800 cm^3^) with an approximate reported cost above $100,000 (£ 90,000) (McKenna et al., 2010). EECP requires the subject to travel to a specific center for use of the device, and to receive technical assistance to operate. Thus, EECP is primarily reserved for medical clinics and would most likely not have a role in the day to day therapeutic management for diabetes. Guidelines for consideration and potential risks and requirements have been published by Lin et al. (2020).

### Enhanced External Counterpulsation and Diabetes

Enhanced External Counterpulsation has been used in subjects with abnormal glucose tolerance. Martin et al. showed improved markers of glucose tolerance including markers of improved insulin sensitivity after 7 weeks of treatment (five one hour session per week) (Martin and Braith, 2012; Martin et al., 2012, 2014). These changes are attributed to improve NO bioavailability and NO mediated glucose uptake (Martin et al., 2012). Sardina et al. found that EECP significantly decreases fasting plasma glucose, post-prandial glucose at 120 min, and HgA1C in patients with T2D (Sardina et al., 2016a). In a longer term study in the same population they found decreased advanced glycation products (AGE) and receptors of advanced glycation products (RAGE) up to 6 months (Sardina et al., 2016b). These findings support a beneficial effect of EECP in the management of diabetes. These data support the hypothesis that PSS has therapeutic potential in the management of diabetes. Unfortunately, due to the need for supervised use, non-portability and cost EECP it cannot be done at home on daily basis as a routine therapeutic strategy.

## Whole Body Vibration

### Whole Body Vibration (WBV) for Pulsatile Shear Stress

Whole body vibrations (WBV) are mechanical oscillations of any frequency which are transferred to the human body. The early physiological effects of body vibration where described by a Swedish gymnast, physician, and inventor Gustav Zander (1835–1920) and almost simultaneously Jean-Martin Charcot (1825–1893) in 1892 described the use of the “vibratory chair” in the treatment of various neurological diseases including Parkinson’s (Goetz, 2009). In 1895, Dr. John Harvey Kellogg (the inventor of Kellogg’s Corn Flakes) was the next inventor to utilize vibration technology for health and wellness, he patented the “Kellogg Chair” (Kellogg, 1908). In the 1960 the Russian space program used WBV as a way to simulate weight bearing loads for their cosmonauts while training and rehabilitation before, during, and after outer space missions. Parallel to the latter, scientific literature in the United States began describing the effects of WBV on central hemodynamic changes, ventilation, and behavioral effects (Hoover et al., 1961; Young et al., 1965; Shoenberger, 1972).

The mechanical oscillations for WBV are performed using a platform which moves in either the linear/vertical direction (up and down) or the pivotal/oscillatory (see-saw) motion of the body (Cardinale and Wakeling, 2005; Rittweger, 2010). The subjects are either standing (with various degrees of knee flexion) or in seated postures. The range of frequencies reported for WBV are 720 to 3,600 cpm (12–60 Hz) and displacements from <1 to 10 mm with Gz + 1.5 mt/sec^2^. Frequencies below 10 Hz with a accelerations of Gz + 1.5 mt/sec^2^ and higher have been shown to induce significant discomfort profiles (Zhou and Griffin, 2014; Huang and Zhang, 2019). There are a wide variety of protocols used with regards to frequencies, amplitudes, and exposure time, depending on the studied condition, additionally the great majority of studies exploring the effects of WBV utilize a structured exercise performed on the WBV platform. WBV has evolved primarily as sports training adjunct in sports medicine, as a method to improve muscle power. The effects of WBV have been summarized in systematic reviews in terms of outcomes for specific populations such as; improvements in bone mineral density in post-menopausal women (Robinson et al., 2016), leg muscle strength and balance improvement in elderly, balance and gait in patients with Parkinson’s disease multiple sclerosis, stroke, improved balance and gait speed in the elderly, and walking performance following stroke (Fischer et al., 2019) improved functional exercise capacity and quality of life in chronic obstructive pulmonary disease, improved muscle strength bone mineralization and balance in children with Down’s syndrome (Saquetto et al., 2018). WBV increases skin blood flow (Johnson et al., 2014; Robbins et al., 2014; Games et al., 2015), and improves endothelial function in an elderly population (Aoyama et al., 2019). Non-athletic commercial claims of weight loss are refuted by systematic reviews which failed to show change in lean body mass in obese subjects (Zago et al., 2018). The approximate cost of WBV platforms ranges from $1,000 to $13,000.

### Whole Body Vibration and Diabetes

In a diabetic animal model (leptin-deficient db/db mice) 12 weeks [1 h per day protocol (*f* = 45 Hz)] of WBV, improved insulin resistance and liver oxidative stress (Liu et al., 2016). In a separate study also using the db/db model, WBV decreased insulin resistance, attenuated hyperglycemia, and attenuated adipocyte hypertrophy in visceral adipose tissue compared to sedentary controls (McGee-Lawrence et al., 2017). Animal studies suggest the WBV may have a role in glycemic control, however, these findings have not been translated to the clinical setting.

In the clinical setting, a randomized interventional trial of 50 non-insulin dependent T2D patients randomized to a supervised exercise program (8 varied exercises performed on WBV platform) which included WBV (*f* = 12–16 Hz) for 12 weeks or control, showed a reduction in HbA1c and fasting blood glucose (del Pozo-Cruz et al., 2014). In this study it was not possible to determine the individual effects of WBV without exercise. A meta-analysis of randomized trials on the effects of WBV in T2D patients also showed that WBV combined with exercise improves glycemic control (Robinson et al., 2016). The acute effects of WBV on T2D elderly women were studied using a single WBV session (*f* = 35 Hz) in which exercise was not incorporated compared to controls. These investigators showed that both control and WBV group had a similar decline in post-intervention glycemia (Pessoa et al., 2018) suggesting that acutely WBV does not independently modify glycemia.

A recent double blind randomized interventional trial comprising of 90 subjects with T2D randomized to 8 weeks of WBV (*f* = 12.5–18.5 Hz) without additional exercise compared to control was unable to show differences in HbA1C, physical function or quality of life in these patients (Dominguez-Munoz et al., 2020). Taken together the above clinical studies suggest that WBV alone without a combine exercise intervention strategy may not have an effect on glycemic control. Questions remain as to whether or not higher or lower frequencies or higher amplitudes may confer benefit for glycemic control, but given the high discomfort profiles documented at lower frequencies and higher amplitudes these may not be appropriate (Huang and Zhang, 2019). Future studies of WBV should provide adherence to recent guidelines on the reporting or WBV parameters, such that comparisons can appropriately be made between and across studies (Wuestefeld et al., 2020).

## Whole Body Periodic Acceleration

### Whole Body Periodic Acceleration (WBPA, pGz) and Pulsatile Shear Stress

Whole Body Periodic Acceleration also known as Periodic Acceleration in the z plane (pGz, in animal models) was fist described by Adams and Sackner over 20 years ago (Adams et al., 2000a, b). WBPA/pGz is the sinusoidal motion of the body in the supine posture in a headward to footward direction using a platform. The frequency of motion of the platform in humans is between 100 and 150 cycles per min (1.6 to 2.5 Hz) with acceleration forces in the z plane Gz ± 0.3 mt/sec^2^. In animal models pigs, rats, mice frequency of operation is 180 (3 Hz), 360 cmp (6 Hz) and 480 cpm (8 Hz), respectively, with the same or similar acceleration forces as used in humans. The movement creates inertial forces which introduce small pulsations to the vasculature, these produce pulsatile shear stress to the vascular endothelium (Sackner et al., 2005a; Uryash et al., 2009). Similarly, to exercise and unlike EECP these pulsations are not synchronized with the cardiac cycle.

Whole Body Periodic Acceleration produces release of endothelial derived nitric oxide in human subjects (Fujita et al., 2005; Sackner et al., 2005b), and animal models (Uryash et al., 2009; Wu et al., 2009, 2012) and genomic upregulation of eNOS occurring over relatively short time period (Wu et al., 2009; Uryash et al., 2015). In addition to eNO, pGz increases expression of the antioxidants; superoxide dismutase, catalase, and total antioxidant capacity (Uryash et al., 2015), along with other endothelial derived anticoagulant, and vasoactive proteins and adrenomedullin (Adams et al., 2005; Martinez et al., 2008). WBPA used in human subjects with heart failure (HF), coronary artery disease (CAD), angina, and peripheral artery disease (PAD) improved measures of quality of life in HF, improved coronary flow reserve in CAD, increased exercise tolerance in angina, and improve walking distance and blood flow in PAD (Kohler et al., 2007; Miyamoto et al., 2011; Rokutanda et al., 2011; Sakaguchi et al., 2012; Takase et al., 2013). Due to the large dimensions (size of a single bed) weight (≈ 225 Kg) cost ($10,000, £9,000) of the human platform, a smaller, simpler, device (a predicate device for WBPA) was designed to meet the needs for portability and user friendliness. A passive simulated jogging device [JD (Gentle Jogger, Sackner Wellness Products, Miami, FL, United States)] was created. The motorized JD, passively moves the feet in alternating motion simulating walking or jogging. With each down stroke of the fore foot a pulsation is added to the circulation, thus inducing pulsatile shear stress. The device can be used in the sitting or supine posture, weighs <6 Kg, has a footprint of 12,300 cm^3^, and an approximate cost of less than $1,000 (£750). JD is well tolerated and simple to operate for home use. Further, JD decreases sedentary behavior induced hypertension (Sackner et al., 2019), and improves short term heart rate variability in both supine and seated posture (Adams et al., 2018).

### Whole Body Periodic Acceleration (WBPA, pGz), Passive Simulated Jogging Device (JD) and Diabetes

#### Cellular Effects of pGz on Oxidative Stress

The effects of pGz on various aspects of diabetes have been studied at the whole animals and cellular level. In a mouse model of type 1 diabetes, oxidative stress (Reactive Oxygen Species, ROS) was measured in isolated cardiomyocytes. Compared to control cardiomyocytes, ROS was increased 340%. pGz performed for 14 days, 1 h daily in these mice decreased ROS production in cardiomyocytes by 50% (Uryash et al., 2015).

#### The Effects of pGz on Diabetes Induced Calcium Dyshomeostasis

We have determined whether or not pGz could modify intracellular Ca^2+^ dyshomeostasis produced by hyperglycemia in type 2 diabetic mice (T2D, C57BL/KsJ-db/db). Diabetic and age-matched controls mice were randomly divided into four groups of animals (*n* = 5 per group): (i) control (CONT), (ii) control pGz (pGz), (iii) Type 2 diabetic mice (T2D), and (iv) Type 2 diabetic mice treated with pGz (T2D-pGz). pGz treatment was performed on unanesthetized, restrained mice using the pGz platform (1 h per day *f* = 480 cpm and Gz ± 3.0 mt/sec^2^) for 14 consecutive days for both diabetic and controls. Single ventricular cardiomyocytes were isolated as previously described (Lopez et al., 1983; Eltit et al., 2013) and intracellular [Ca^2+^]_d_ determined in isolated cardiomyocytes by double-barreled selective microelectrodes (Mijares et al., 2014).

**FIGURE 2:**
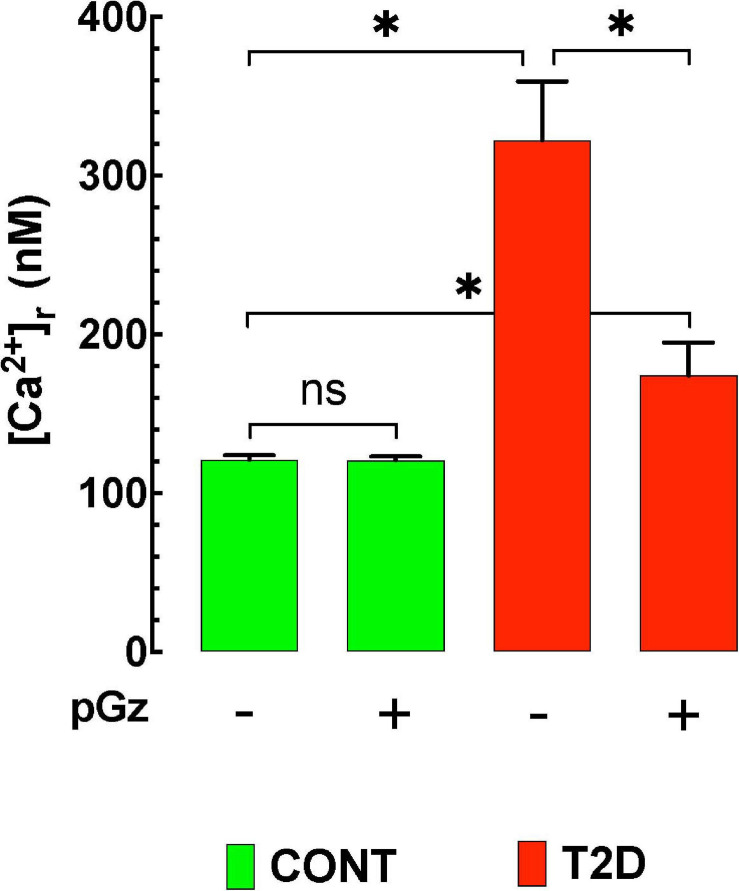
The effects of pGz on diabetes induced cardiomyocyte intracellular Ca^+2^ overload. Intracellular Ca^+2^ is elevated in a mouse mode of type 2 diabetes (T2D). Whole body periodic acceleration (pGz) performed (1 h. per day *f* = 480 cpm and Gz ± 3.0 mt/sec^2^) for 14 consecutive days in both diabetic and controls. pGz did not modify intracellular Ca^+2^ in control animals (CONT), but significantly reduced it in diabetic mice by 46% (*n* = 5 per group). The *X* axis denotes the + (use) or –(absence) of whole body periodic acceleration (pGz). *p < 0.001, ns, not significant).

[Ca^2+^]_d_ was 122 ± 3 nM (*n* = 20) in control polarized cardiomyocytes compared to 322 ± 37 nM (*n* = 17) in T2D cardiomyocytes (*p* ≤ 0.05) (Figure 2). Treatment with pGz for 14 days reduced [Ca^2+^]_d_ by 46% in T2D cardiomyocytes mice (to 174 ± 21 nM, *n* = 20) (*p* ≤ 0.001 compared to untreated age-matched T2D cardiomyocytes). In control cardiomyocytes, pGz did not significantly modify [Ca^2+^]_d_ (121 ± 3 nM, *n* = 21, *p* ≥ 0.05) (unpublished results). The above findings confirm calcium dyshomeostasis in T2D mice, and show that pGz via PSS may be a coadjuvant candidate in preventing and treating calcium overload in diabetic cardiomyocytes.

### The Effects of WBPA and JD on Diabetes

A single 1 h session of WBPA in T2D, reduced serum insulin and increased total and high molecular weight adiponectin, which acts as a key modulator of insulin sensitivity and glucose metabolism (Sakaguchi et al., 2012).

The effects of JD have also been studied in healthy volunteers and T2D subjects in an at home clinical study. The effects were measured using interstitial continuous glucose monitoring (FreeStyle Libre Pro, Abbott, Alameda, CA, United States). There were no attempts to modify diet or usual activities of daily living, all subjects continued to take their usual medications. Subjects were instructed to use the JD at least three times per day for 30 min for 7 days. Compliance with home use of the device was very good (98% of subjects used the device 90 or more minutes per day). Continuous glucose monitoring showed; daily mean glucose, SUM of all glucose values, 24 h glucose area under the curve (24-AUC), were all significantly reduced from baseline values during the 7 day use of JD in both healthy and T2D. Additionally, time above glucose >180 mg/dl, was significantly reduced in T2D (Adams et al., 2020). Taken together these studies on WBPA/pGz and JD supports their beneficial effects on diabetes subjects.

The described methods to induce PSS are varied with respect to frequency, accelerations, and known effects on clinically measured aspects of diabetes. Nearly all of the methods have shown the biochemical effect of NO, anti-inflammatory, fibrinolysis, and improved endothelial function. They are also varied as to subject-device interface and portability. The characteristics of each of the aforementioned interventions to produce PSS, are summarized in Table 1.

**TABLE 1:** Comparison of methods to induce pulsatile shear stress.

	Exercise	EECP	WBV	WBPA	JD
**Mechanical Characteristics**					
Frequency Range (Hz)	2.7–3.0 (Kawabata et al., 2013; Clermont et al., 2019)	1–1.7 (Raza et al., 2017; Lin et al., 2020)	12–60 (Sonza et al., 2015; Rawer, 2020; Spain et al., 2020)	1.7–3.0 (Kohler et al., 2007; Rokutanda et al., 2011)	1.7–3.0 (Sackner et al., 2019, 2020)
Root Mean Square Acceleration (a _RMS_) (m/s^2^	1.5–2.5	N/A	2.1–7.0 dependent on amplitude, f, and location of measurement (Sonza et al., 2015)	2.0–3.5	1.0–3.0
**Biochemical Effect**					
Nitric Oxide Effect	↑	↑	N/A	↑	↑
eNO	↑	↑	N/A	↑	↑
Anti-Inflammatory	+	+	+	+	+
Fibrinolysis	Yes (Womack et al., 2003) Uncertain (Patelis et al., 2016)	Yes (Comerota et al., 1997) No Effect (Arora et al., 2005)	N/A	↑	↑
Intracellular Calcium	↓	N/A	N/A	↓	↓
**Effects on Diabetes**					
Peak- Glucose	↓	N/A	N/A	↓	↓
OGTT (AUC)	↓	↓	N/A	N/A	↓
Time in Glucose Range	Improves	N/A	N/A	N/A	Improves
HbA1C	↓	↓	↓ or N.C	N/A	N/A
Improved Insulin Sensitivity	+	N/A	N/A	+	N/A
Improves Endothelial Dysfunction	+	+	+	+	+
**Subject Usage**					
Requires Cognitive/Physical Ability	YES	YES	YES	NO	NO
Device Portability	N/A	NO	NO (except commercial devices)	NO	YES
Device Weight	N/A	250 Kg	15–160 Kg	225 Kg	<6 Kg
Device Home Use	N/A	NO	YES	YES	YES
Weight Management	YES	NO	NO	NO	NO
Change in Oxygen Consumption (ml/kg/min)	Variable increase proportional to increase in cardiac output	47% in 1st 15 min of use. No change at end of a session of EECP (Ahlbom et al., 2016)	36% compared to standing, <2 METS (Fares et al., 2016)	N/A	12–15% in both supine and seated posture. No change at end of JD. 1.5METS (Sackner et al., 2020)

*EECP, enhanced external counterpulsation; WBV, whole body vibration; WBPA, whole body periodic acceleration; JD, passive jogging device; eNO, endothelial derived nitric oxide; OGTT, oral glucose tolerance test; AUC, area under the curve. Time in Glucose Range = amount of time of plasma glucose <180 mg/dl in a 24 h period. ↑ = increase ↓ = decrease, N/A = not available, N.C. = no change, + = positive effect.*

## Limitations

There are limitations in this present narrative review which must be acknowledged. The review has focused on 4 of the most common methods for sustained PSS, however, there are other methods such as vibro percussion (Hoffmann and Gill, 2012), and passive cycling or limb movement (Trinity et al., 2015; Hosseini et al., 2019), which may also have potential for diabetes therapeutics, but no studies are available on diabetics. Exercise is at the cornerstone of diabetes therapy, and the recommendations for exercise in T2D (Colberg et al., 2016; Piercy et al., 2018) provide guidance, however, we did not address in this review the optimum exercise prescription “dose” (duration, frequency, and intensity) the latter is a topic which remains largely unresolved (Durand and Gutterman, 2014; Pedersen and Saltin, 2015; Simon, 2015; Wasfy and Baggish, 2016; Radak et al., 2017; Eijsvogels et al., 2018; Leggio et al., 2018; Ried-Larsen et al., 2018; Herold et al., 2019; Pedersen, 2019; Powers et al., 2020). It is also clear from the literature that any exercise is better than none, and that cumulative effects are better than single bouts. Recent Meta-analysis on the effects of exercise in T2D subjects on vascular endothelial function, have concluded that exercise training in general increases flow mediated vasodilation (FMD) particularly aerobic and combined aerobic and resistance exercise (Qiu et al., 2018), and low to moderate (50–70% of heart rate max, or 60–65% oxygen consumption) intensity, increased FMD more than high intensity (Lee et al., 2018). We have also not discussed the value of interval training or high intensity interval training (HIIT) which potentially require less time commitment from the patients but greater subject engagement and fitness. A recent 1 year randomized controlled trial in patients with T2D showed that HIIT improved peripheral arterial stiffness and indices of distensibility (Magalhaes et al., 2019). In the recently reported D2FIT study (a 1 year randomized controlled study in T2D), non-exercise, moderate continuous training, resistance training (RT) and HIIT with RT, it appears that regardless of improvement in cardio respiratory fitness (peak oxygen consumption) with exercise, all interventions improved carotid intima-media thickness and pulse wave velocity markers of vascular health (Hetherington-Rauth et al., 2020). Further, a recent review on the inter-individual variability in the therapeutic response of blood glucose to exercise has identified knowledge gaps with regard to exercise dose, meal timing, anti-hyperglycemic drugs which must be addressed for optimizing therapeutic benefit of exercise (Solomon, 2018). It is clear that the overall goal of exercise in diabetes management is to decrease physical inactivity, exercise and the afore mentioned interventions all decrease physical inactivity. Higher levels of physical activity are associated with lower mortality rates in T2D (Geidl et al., 2020). Finally, we have not addressed adherence and barriers to exercise or the aforementioned interventions. Reduced adherence to physical activity guidelines in diabetics are associated with daily smoking, obesity chronic kidney disease and poor health and the latter associated with barriers such as pain, fatigue, physical limitations and hospitalizations (Barker and Eickmeyer, 2020; Martinez-Harvell et al., 2020). The interventions discussed in this review are not intended to take precedent over any exercise regimen, they are targeted to the population of subjects who cannot or will not exercise.

## Conclusion

Pulsatile Shear Stress, to the vascular endothelium is clinically feasible and a potential therapeutic intervention in the management of diabetes. In subjects for whom physical exercise is not possible both EECP and JD are strategies which are easily used. The vasculoprotective, antioxidant, anti-inflammatory, and glucose lowering, effects of PSS can be harnessed to improve management and outcomes from diabetes and other chronic conditions. Given the magnitude of physical inactivity worldwide, PSS via non-invasive methods are plausible stop gap alternatives for those who can’t or won’t exercise. Further studies are needed to ascertain the optimum duration of use of these devices, and additional mechanistic insights.

## Author Contributions

JA conceived and wrote the initial manuscript. AU performed the laboratory experiments and edited the manuscript. JL performed the experiments and co-wrote the manuscript. MS co-wrote and edited the manuscript. All authors contributed to the article and approved the submitted version.

## Conflict of Interest

MS and JA draw no salary from Sackner Wellness Products LLC a company which has a patent on a passive jogging device. MS owns 80% and JA 20% of the domestic and foreign patents. The remaining authors declare that the research was conducted in the absence of any commercial or financial relationships that could be construed as a potential conflict of interest.
